# Reactive oxygen species accumulation is synchronised with growth inhibition of temperature-sensitive *recAts polA Escherichia coli*

**DOI:** 10.1007/s00203-022-02957-z

**Published:** 2022-06-16

**Authors:** Akihiro Kaidow, Noriko Ishii, Sinngo Suzuki, Takashi Shiina, Hirokazu Kasahara

**Affiliations:** 1 Department of Biology, School of Biology, Tokai University, Sapporo, 005-8601 Japan; 2 Department of Bioscience and Technology, School of Biology, Tokai University, Sapporo, 005-8601 Japan; 3 Department of Molecular Medicine, School of Medicine, Tokai University, Isehara, 259-1193 Japan

**Keywords:** *recAts polA* lethality, Redox molecular chaperon, Hsp33, ROS, Oxidative damage, SOS response

## Abstract

When combined with recombinase defects, chromosome breakage and double-strand break repair deficiencies render cells inviable. However, cells are viable when an SOS response occurs in *recAts polA* cells in *Escherichia coli*. Here, we aimed to elucidate the underlying mechanisms of this process. Transposon mutagenesis revealed that the *hslO* gene, a redox chaperone Hsp33 involved in reactive oxidative species (ROS) metabolism, was required for the suppression of *recAts polA* lethality at a restricted temperature. Recently, it has been reported that lethal treatments trigger ROS accumulation. We also found that *recAts polA* cells accumulated ROS at the restricted temperature. A catalase addition to the medium alleviates the temperature sensitivity of *recAts polA* cells and decreases ROS accumulation. These results suggest that the SOS response and *hslO* manage oxidative insult to an acceptable level in cells with oxidative damage and rescue cell growth. Overall, ROS might regulate several cellular processes.

**Supplementary Information:**

The online version contains supplementary material available at 10.1007/s00203-022-02957-z.

## Introduction

The ability of bacteria to rapidly grow comes at the price of exposing its chromosomes to DNA damage; however, when DNA damage is sensed, cell division is delayed by the SOS response (Simmons et al. 2008). In particular, the mitotic inhibitor SfiA inhibits FtsZ, which constitutes the cell division machinery. However, unlike eukaryotic cells, a check point (Parrilla-Castellar et al. 2004) for blocking chromosome initiation in bacterium has not yet been identified. The RecA protein of *Escherichia coli* plays a crucial role in homologous recombination and repair (Kowalczykowski et al. 1994), functioning both as a recombinase and coprotease. Defects in DNA polymerase I (pol I) function, such as the *polA25* mutation, accumulated nicks, and gaps owing to failures when processing Okazaki fragments, can cause DNA damage. When a replication fork encounters a discontinuity in a template, it results in a double-strand break (DSB) in *E. coli* chromosomes. Recombination-dependent DNA replication repairs collapsed replication forks (Kogoma 1997).

Synthetic lethality, in which the combined knockout of two non-essential genes or reactions is lethal, has direct applications in understanding cellular processes. Both *recA polA* and *recB polA* double mutants are inviable owing to deficiencies in DSB repair (Monk and Kinross 1972). LexA protein, an SOS repressor, regulates the expression of SOS genes in response to DNA damage. A *lexA71* mutation completely inactivates the LexA repressor, thereby de-repressing the LexA regulon (Walker 1996). This *lexA* mutation suppresses the lethality of the *recA polA* double mutant (Cao and Kogoma 1995). This inducible system has been designated to suppress *recA polA* lethality (Srp). A constitutive SOS response is crucial for the Srp pathway, requiring a mutation in the *sfiA* gene to allow cell division. Thus, factors involved in the Srp pathway presumably include not only suppressors of *polA* and *recA* mutations but also damage response and signal factors related to DNA damage repair until cell division.

*hslO*, encoded by Hsp33, is a redox molecular chaperone that protects organisms against oxidative stress, leading to protein unfolding. The absence of this function results in cells being sensitised to hydrogen peroxide (Jakob et al. 1999). Activation of HslO is triggered by the oxidative unfolding of its redox-sensor domain, which classifies HslO as a member of a recently discovered class of chaperones that require partial unfolding for full chaperone activity (Reichmann et al. 2012).

*E. coli* frequently encounters reactive oxygen species (ROS) in its environment, typically in the form of H_2_O_2_ generated as a by-product of metabolic activities. Recent studies have drawn attention to the contributions of stress; it stimulated the accumulation of ROS (Kohanski et al. 2007) and elicited cell death after lethal treatments (Hong et al. 2019). However, the accumulation of ROS itself is bacteriostatic rather than bacteriocidal (Imlay 2015). It has been reported that thymine starvation leads to the accumulation of both single-stranded DNA regions and intracellular ROS (Hong et al. 2017). Hong et al*.* also reported that lethal action is derived from the stimulation of the self-amplifying accumulation of ROS, which overwhelms the repair of primary damage (Hong et al. 2019). Cells face replicative stress during growth (Zeman and Cimprich 2014); however, ROS, like NO in the nervous signal system, has a short half-life owing to its high reactivity. Therefore, ROS would function as an effector because of their high reactivity and serve as both a stress marker (Nikitaki et al. 2015) and signal factor (Decros et al. 2019).

The relationship between DNA damage and ROS production has remained unclear; therefore, in this study, we explore the growth of *recA polA* cells in the presence of ROS and elucidate the pathways involved. By investigating ROS as a determinant for cell growth, the intricacies of redox signalling in *E. coli* can be further understood.

## Materials and methods

### *E. coli* strains, phenotypes, and culture media

The *E. coli* strains used in this study are listed in Table 1. Strains were constructed using phage P1*vir* mediated transduction (Lennox 1955; Miller 1992). Cells were grown at 30 °C in M9 minimal salts–glucose (M9G) media supplemented with casamino acids (CAA) (0.2%; Difco Laboratories, Detroit, MI, USA), thymine (2 μg/mL), thiamine (0.1 μg/mL), appropriate amino acids (50 μg/mL), i.e. arg, thr, leu, trp, his, and pro (M9GCAA medium), as well as ampicillin (20 μg/mL), kanamycin (55 μg/mL), spectinomycin (40 μg/mL) and streptomycin (100 μg/mL).

**Table 1:** *Escherichia coli* strains used in this study

Strain	Relevant genotype	Source, reference, or construct
AQ1023	*sfiA11 pyrD*::Tn*5*	As GC2277 This laboratory
AQ2153	*lexA71*::Tn*5*	As K-196 This laboratory
AQ6039	*polA12*	As MM383 This laboratory
AQ6471	*malF-3089*::Tn*10*	This laboratory
AQ6730	KL268 *recA200*	This laboratory
AQ8534	*polA25*::*spc zih-35*::Tn*10*	Cao et al. (1993)
AQ8846	*thy*^+^	As AB1157 but Thy^+^ (Asai et al. 1994)
AQ10452	*sfiA11 pyrD*::Tn*5*	AQ8846 × P1.AQ1023 – > Km^r^, UV^r^
AQ10458	As AQ10452 but Km^s^	Km^s^ (spontaneous)
AQ10529	*srp-529 mini*Tn::*10*	This work (experimental procedure)
AQ10546	AQ10458 *recA200*	AQ10458 × AQ6730 – > His^+^, UV Ts
AQ10549	AQ10546 *polA25 zih-35*::Tn*10*	AQ10546 × P1.AQ8534 – > Tc^r^, Ts
AQ10553	As AQ10549 but Tc^s^	Tc^s^ (Bochner selection)
AQ10724	*polA12 srp-529*	AQ6039 x P1.AQ10529 – > Tc^r^
AQ10865	*lexA51 malB*::Tn*9*	This laboratory
AQ11294	*Δsrp*::*Km*	This work (Experimental procedure)
AQ11351	AQ10553 *lexA71*	AQ10553 × P1.AQ2153 – > Km^r^, Tr
AQ11369	*ΔhslO*::*Km*	This work (Experimental procedure)
AQ11466	AQ11351 *lexA51*	AQ11351 × P1.AQ10865 – > Cm^r^, Km^s^
AQ11474	AQ10546 *lexA71*	AQ10546 × P1.AQ2153 – > Km^r^
AQ11527	AQ11474 *malF*::Tn*10*	AQ11474 × P1.AQ10865 – > Cm^r^, Km^s^
AQ11544	AQ11466 *malF*::Tn*10*	AQ11466 × P1.AQ6471 – > Tc^r^, Cm^s^,
AQ11580	AQ11527 *Δsrp*::*Km*	AQ11527 × P1.AQ11294 – > Km^r^
AQ11582	AQ11527 *ΔhslO*::*Km*	AQ11527 × P1.AQ11369 – > Km^r^
AQ11588	AQ11466 *ΔhslO*::*Km*	AQ11466 P1.AQ11369 – > Km^r^
AQ11591	AQ11544 *Δsrp*::*Km*	AQ11466 × P1.AQ11294 – > Km^r^
AQ11594	AQ11466 *Δsrp*::*Km*	AQ11466 × P1.AQ11294 – > Km^r^
AQ11735	AQ10458 *polA25 zih-35*::Tn*10*	AQ10458 × P1.AQ8534 – > Tc^r^, UV^s^
TK3276	AQ10549 *ΔhslO*::*Km*	AQ10549 × P1.AQ11369 – > Km^r^
TK3072	AQ10549 pMW119	AQ10549 × *p*MW119 – > Ap^r^
TK3075	AQ10549 pEXsrpC	AQ10549 × *p*EXsrpC – > Ap^r^
TK4166	TK3276 pMW119	TK3276 × *p*MW119 – > Ap^r^
TK4169	TK3276 pEXsrpC	TK3276 × *p*EXsrpC – > Ap^r^
TK4558	AQ11588 pMW119	AQ11466 × *p*MW119 – > Ap^r^
TK4559	AQ11588 pEXsrpC	AQ11466 × *p*EXsrpC – > Ap^r^

AQ8846 genotype: *F- argE3 his-4 leuB-6 proA2 thr-1 thi-1 rpsL31 galK2 lacY1 mtl-1 supE44*

*Tr* temperature-resistant growth at 42 °C, *Ts* temperature-sensitive growth at 42 °C, *Tc*^*r*^, *Km*^*r*^, *Cm*^*r*^ and *Ap*^*r*^ resistance to tetracycline, kanamycin, chloramphenicol, and ampicillin, respectively; *Tc*^*s*^, *Km*^*s*^ and *Cm*^*s*^ susceptibility to tetracycline, kanamycin, and chloramphenicol, respectively; *UV*^*s*^ sensitive to UV, *UV*^*r*^ resistant to UV

### Cultivation and sampling methods

Two and 15 mL of M9GCAA liquid medium were placed in test tubes and 100 mL Erlenmeyer flasks, respectively, and cultured aerobically at either 30 °C or 42 °C. Cells were inoculated with 1/100 volume of cells grown overnight on M9GCAA broth.

In shift-up experiments, cells were cultured in M9GCAA medium until OD_600_ = 0.1. Cells were then divided into two to four equal portions for the addition of reagents. After the indicated treatments, the cell cultures were measured for OD_600_, DNA content, and ROS analysis every 2 h from 0 to 16 h. For the time-course experiment, typical sample volumes were 600 μL for OD_600_, 200 μL for DNA content, and 4 μL for ROS analysis.

In inoculation experiments, cells without or with a predetermined menadione concentration were incubated at 120 rpm for 16 h at either 30 °C or 42 °C.

### Determination of survival fractions and cell recovery

For RV determination, after incubation in M9GCAA medium overnight at 30 °C, the cells were diluted in M9 medium without a nutrient source (M9B), and then plated on M9GCAA plates supplemented with appropriate antibiotics and incubated for 16 h at either 30 °C or 42 °C. 10^3^, 10^4^, and 10^5^ particles were spread on the plates, and the viability was determined from the number of colonies grown on each plate.

In the complementation test, the RV was determined by comparing the number of colonies under restrictive temperature with the permissive temperature.

In the spot method, 2 μL of the diluted culture medium containing the indicated number of particles was smeared on the plate by spotting, incubated at 30 °C or 42 °C for 16 h. Grown cells on agar were collected from the surface of the spotted agar by wiping with a sterilised tip, suspended in 20 μL of M9B.

### Southern hybridisation and sequencing

Chromosomal DNA was extracted using Wilson’s method (Wilson 2001). Southern hybridisation was performed as described by Sambrook et al. (1989)*.*

DNA sequences were determined using a commercially available sequencing kit, Sequenase Version 2.0 (USB Corp.). Computer analysis of the DNA sequences was performed using GCG sequence analysis software, version 3.0. Sequences of the long PCR products were determined as described by Suzuki et al. (2017). *hslO* gene in KEGG database, ecj: JW5692 or eco: b3401.

### Isolation of the *srp-529*::mini*Tn10* allele

An exponentially growing culture of MC1000 carrying the plasmid pNK2883 (Kleckner et al. 1991) was incubated at 30 °C with IPTG (10^–4^ M) for 30 min. Cells were then infected with P1*vir*, and the resulting lysate was used to transduce AQ8695 (Cao and Kogoma 1995) for tetracycline resistance (Tc^r^). All Tc^r^ colonies were screened for temperature-sensitive growth using replicate plating. Temperature-sensitive cells were then infected with P1*vir*. The resultant P1 phage lysate was used to transduce AQ8695 again for Tc^r^. Three colonies grew at 30 °C but failed to grow at 42 °C. We designated this mutated allele *srp-529*::miniTn*10* (Kogoma 1997), confirmed by Southern hybridization (Sambrook et al. 1989).

### Cloning *srp-529*::miniTn*10*

*p*NK2805 (Kleckner et al. 1991) was integrated into the genome of strain AQ10724 (*polA12 srp-529*::miniTn*10*) using in vivo homologous recombination on Ap plates at 42 °C. Chromosomal DNA was then isolated, subsequently digested with *Sal*I, and self-ligated. To obtain the wild-type *srp* DNA fragment, *l* clone 620 of the Kohara library was digested with *Bam*HI. The resulting 3.4 kb *srp* fragment was cloned into *p*BS^A^ (pBluescript *Afl*III site disrupted derivative).

### Construction of *srp* mutants

A low copy number *srp* operon plasmid, *p*AQ10929, was digested with both *Afl*III and *BstE*II. A digested 2.0 kb *srp* DNA fragment was replaced with a *Bam*HI kanamycin-resistant (Km^r^) fragment from plasmid *p*NK2804. The resulting Km^r^ plasmids were linearised with *Sal*I and *Sac*I. AQ5563 (*recD1104*) cells were transformed with the linearised plasmids. Transformants were selected for Km^r^. These resultant *Δsrp* mutations were transduced into AQ634 (Cao et al. 1993), resulting in AQ11294. The *srpC*/*hslO* deletions were constructed as described above, except that pAQ10929 was digested with *Bst*EII. The 0.7 kb *srpC* DNA fragment was replaced as described above. The resultant transduced strains were termed AQ11369.

### Plasmid constructions

The 3.4 kb *srp* DNA fragment was cloned into the *Bam*HI site on *p*HSG576 (Asai et al. 1993) and *p*BS^A^. The resultant plasmids were termed pAQ10929 and *p*AQ10917, respectively. The *srp* DNA fragments shown in Fig. S1 are *p*SRO1 (*Bam*HI-*Bam*HI), *p*SRO*Δ*L1 (*Stu*I-*Bam*HI), *p*SRO*Δ*L12 (*Afl*III-*Bam*HI), *p*SRO*Δ*P1 (*Nsi*I-*Bam*HI), *p*SrpC (*Bsm*I-*Bam*HI), and *p*SRO*Δ*hslO (*Bam*HI-*Bst*EII and *Bst*EII-*Bam*HI). To construct the 5′-end deletion plasmids (*p*SRO*Δ*L1, *p*SRO*Δ*L12, *p*SRO*Δ*P1, and *p*SrpC), intermediate plasmids with a *Bam*HI site at the 5′ *srp* DNA fragment as a unique site were digested with *Bam*HI and either *Stu*I, *Afl*III, *Nsi*I, *Bsm*I, or *BstE*II, and then self-ligated. The resultant deletion plasmids were digested with *Eco*RI (on the vector) and *Sph*I or *Pst*I (on *srp*, Fig. S1), and *EcoRI-SphI/PstI* fragments were transferred into *p*AQ10929. *p*SRO*Δ*hslO was constructed by *p*AQ10929 digestion with *Bst*EII. The resultant plasmids were digested with *Eco*RI and *Sal*I and cloned into *the Eco*RI and *Sal*I sites of *p*HSG576. To prevent transcription from the plasmid vector, an Spc^r^ cassette that possessed T4 translation–transcription termination signals at both ends (Prentki and Krisch 1984) was introduced into the *Sma*I site. *p*HSG*/spc* was the *p*HSG576 derivative carrying the Spc^r^ cassette. All DNA manipulations were carried out using T4 DNA polymerase or Klenow fragments (New England Biolabs Inc.), as described by Sambrook et al. (1989).

To construct a *lacZ* leader-*hslO* expression plasmid (*p*EX*srpC*), *hslO/srpC* DNA fragments were amplified by PCR with TAKARA Ex *Taq* (Takara Co., Ltd.). For the amplification of *hslO*, the primers used were 5′-AAACTCGAGATGATTATGCCGCAACATGA-3ʹ and 5′-AAAGGATCCTTAATGAACTTGCGGATCTG-3ʹ. *p*AQ10917 was amplified by 15 cycles at 94 °C: 1 min, 50 °C: 1 min, and 72 °C: 2 min. The amplified *srp* DNA fragments were digested with *Xho*I and *Bam*HI and cloned into the *Xho*I and *Bam*HI sites of the *p*MW119 (Takara Co., Ltd.). The *srp* fragment containing plasmids was colour-selected with X-gal. The plasmids were digested with *Hind*III and then self-ligated.

### Flow cytometry analysis

For ROS analysis by flow cytometry, staining was performed according to the study by Manoil and Bouillaguet (2018) but without sonication and fixation of the cell culture. Cell cultures (4 μL) at the indicated times were mixed with 12.5 μM CellRoxDeep Red (16 μL), diluted with M9 medium without organic nutrients (M9B), and stained for 30 min at 25 °C. Stained cells (20 μL) were then diluted in M9B (200 μL). Except for cells from an agar plate, we stained cells with CellRoxDeep Red alone. We then used a Becton Dickinson Accuri C6 (Becton, Dickinson and Company, Ann Arbor, MI, USA) with a 640 nm laser. First, we analysed the cell culture for the gate derived from cell particles. We used identical side scatter signal/forward scatter signal (FSC) gates, designated as P3 (Fig. S2a), and collected 50,000 events. For cells recovered from an agar plate, P4 (Fig. S2b) was used. In our experiments, the rate of events was less than 2,500 events per second. To analyse the acquired data, we used the C6 software, version 1.0.264.21. Each sample was plotted as a histogram vs. the red channel (FL4-A with 675 ± 15 nm filter) or ROS levels (fluorescence, channel FL4-A) as either autofluorescence by the green channel (FL-1A) or as a function of the cell size (as FSC above).

For double staining, we used a P4 gate. In the double staining, single and double staining with each fluorescent dye was performed, and CellRox DeepRed staining was performed as described above. Syber Green I staining was performed according to the manufacturer recommended concentration: M9B (16 μL) containing a 1.25× concentration of SYBR Green I was added to the bacterial recovery solution (4 μL), resulting in 1/8000 dilution of the original concentration. For double staining, CellRox staining solution (16 μL) containing a 1.25× concentration of Syber Green I was added with bacterial recovery solution (4 μL) for 30 min. Unstained (black) CellRox is shown in Fig. S2c. To analyse *E. coli* particles with nucleic acids, unstained (black) and Syber Green I stained (red) were compared (Fig. S2d), and M-1 was set as the gate for the nucleic acids. The leakage of the unstained particles into the M-1 was less than 0.1%. The amount of ROS was analysed based on the FL-4A as particles with nucleic acids. DNA content analysis was carried out according to Ferullo et al. (2009).

To determine the number of particles in cultures, the BD Cell Viability Kit (Becton, Dickinson and company, 335925) was used following the manufacturer’s procedure.

### Statistical analysis

Calculation of means was performed using Microsoft Excel, and the standard error of the mean (SEM) was calculated with the STDEV.P function.

## Results

### *hslO* is involved in the suppression of temperature sensitivity in *recA*ts *polA* cells

The *recAts polA* temperature sensitivity was suppressed by the introduction of the *lexA*(Def) mutation. This suppressive pathway, designated as the Srp pathway, has not previously been reported. Therefore, we attempted to identify the genes involved in the Srp pathway. Transposon mutagenesis was carried out in MC1000 cells with pNK2883 (Kleckner et al. 1991) using the induction of transposase with IPTG, and mutagenised cells were used to prepare P1 lysates. AQ8695 (*recA200 polA25 lexA71 sfiA11*) (Cao et al. 1993) cells were infected with these P1 lysates to transduce miniTn*10* mutagenised genes. Among the 1,523 Tc^r^ transductants, three colonies were temperature sensitive at 42 °C. One colony was isolated, and we designated this mutated allele *srp-529*::miniTn*10* (Kogoma 1997).

The mutant gene was cloned via homologous recombination. AQ6039 (*polA12*) (Monk and Kinross 1972) cells were transduced with P1 lysate from AQ8695 with *srp-529*::miniTn*10* cells, resulting in AQ10724. *polA12* cells failed to maintain the ColE1 plasmid at 42 °C unless it was integrated into the host chromosome with selective pressure for an antibiotic resistance marker on the plasmid. AQ10724 (*polA12 srp-529*) cells were transformed with *p*NK2805, which was integrated into the genome via homologous recombination at 42 °C. The *srp-529* mutated gene fragment was cloned by the self-circularization of chromosomal DNA from integrated cells.

**Fig. 1:**
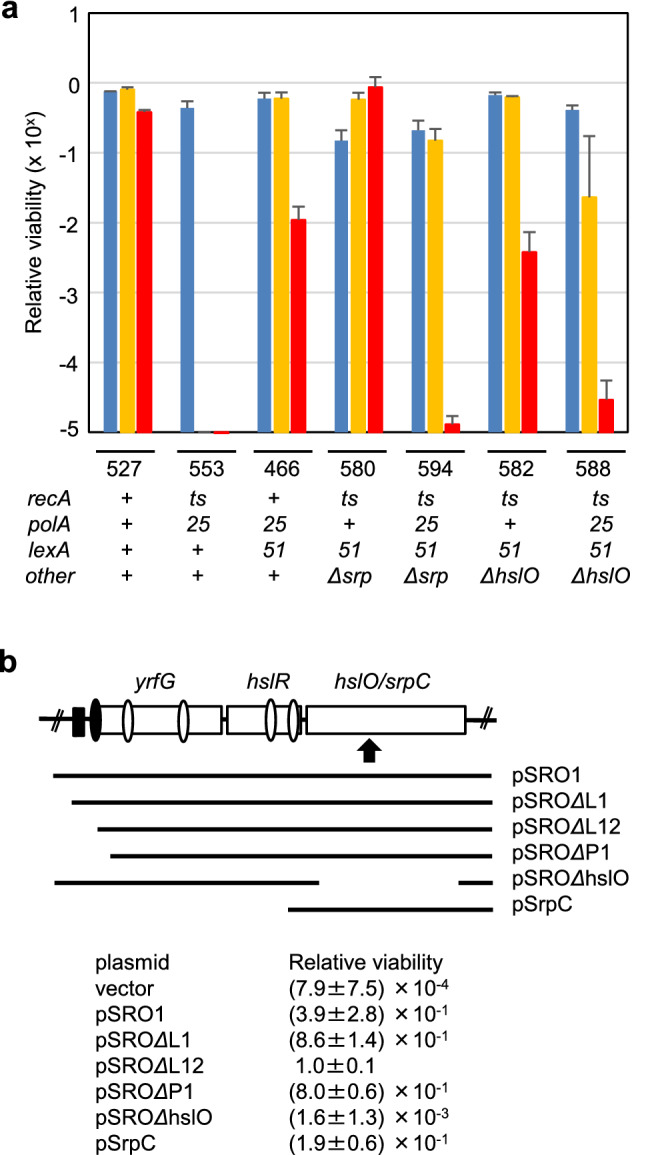
*hslO* genes are required to suppress the temperature sensitivity in *recAts polA* cells. **a** Plating efficiency of *recAts polA* strains. The plating efficiencies were determined as the ratio of viable cells to the number of particles at the indicated temperatures on the M9GCAATT plates: 30 °C (blue), 42 °C (orange), and 44 °C (red). Bars are shown as standard error of the mean (*n* ≥ 3, SEM). Below, 3 figure numbers are corresponding to their last 3 figure number of the strain. The genotypes were as follows: 527: AQ 11527 (*recA200 lexA51*), 553: AQ10553 (*recA200 polA25*), 466: AQ11466 (*recA200 polA25 lexA51*), 580: AQ11580 (*recA200 lexA51 Δsrp*) 594: AQ11594 (*recA200 polA25 lexA51 Δsrp*) 582: AQ11582 (*recA200 recA200 polA25 lexA51 ΔhslO*) and 588: AQ11588 (*recA200 polA25 lexA51 ΔhslO*). **b** Complementation of *recAts polA lexA51 Δsrp* cells (AQ11591) by low copy plasmids carrying different fragments of the *srp* operon. The gene organisation of this region is shown. The putative *srp* operon genes are indicated as open boxes. Black boxes upstream of *the yrfG* were LexA-like sequences. The arrowhead indicates the position of the miniTn*10* insertion of the *srp-529* mutation. Double slash indicates omission of chromosome region. Promoters in the *srp* region are shown as ovals: P1 promoter (black oval). The restriction endonuclease cleavage sites were as follows: B; *Bam*HI, St; *Stu*I, A; *Afl*III, N; *Nsi*I, Bsm; *Bsm*I and Bs; *Bst*EII. DNA fragments harboured on individual plasmids are represented by bars with their plasmid names. Below, the name of each plasmids, the relative viability of the cells harbouring each plasmid (42 °C/30 °C) are shown as standard error of the mean (*n* ≥ 3, SEM)

The DNA sequence 5′-TATCACCTTCCAGACCAACTACGCCCTGAT-3′ was obtained from the cloned *srp-529*::mini Tn*10* DNA fragment, followed by the IS*10* sequence. A search of the GenBank and KEGG database revealed that the sequence from the *srp-529* mutation (designated as the *srpC* gene) corresponded to *hslO*. Additionally, two more genes were located upstream of *hslO/srpC*. These three genes encode YrfG (Kuznetsova et al. 2006), HslR (Korber et al. 2000), and HslO (Jakob et al. 1999), and are likely to constitute a putative operon (Blattner et al. 1997). In addition to putative promoters for the gene encoding YrfF protein (Karp et al. 2018), LexA box (Simmons et al. 2008)-like sequences were found 36- and 116-bp upstream of the *yrfG* gene (Fig. S1). We propose that the Srp pathway may be under the control of the *lexA* gene (Cao and Kogoma 1995). To further investigate this, we analysed the promoter activities of these genes. We identified five promoters in the *srp/hsl* locus. Upstream of the *yrfG* gene, the most proximal promoter (P1 promoter: *yrfG*p) is adjacent to two *lexA* box-like sequences (Fig. 1a), and the P1 promoter was weakly regulated by *lexA* (Fig. S3), which corresponds to the proximal promoter transcribed by σ^70^ (Wade et al. 2006; Karp et al. 2018). This supports the idea that the Srp pathway is induced by the SOS response. To avoid the complexity of insertion mutations, we constructed a *hslO* gene deletion mutation (*ΔhslO*) and an *srp/hsl* operon gene deletion mutation (*Δsrp*). We examined the effects of these *ΔhslO* and *Δsrp* mutations on plating efficiency at various temperatures in *recA polA lexA*(Def) cells (Fig. 1a). The plating efficiency for the AQ10553 (*recA200 polA25*) cells was 10^–5^ at 42 °C and 10^–6^ at 44 °C. In contrast, the plating efficiencies of the *recA polA* lethality suppressed cells (AQ11466) were 0.47–0.73 and 0.005–0.017 at 42 °C and 44 °C, respectively. The suppression of temperature sensitivity by the Srp pathway was blocked at 44 °C in both AQ11594 (*Δsrp* derivative) and AQ11588 cells (*ΔhslO* derivative). The plating efficiency of these cells was reduced to approximately 10^–5^ at 44 °C. These results indicate that the Srp pathway is blocked by the *ΔhslO* mutation. Therefore, we attempted to determine the minimum *srp/hsl* DNA fragment required for the Srp pathway by complementation tests in AQ11591 (*recA200 polA25 lexA51 Δsrp*) cells.

AQ11591 cells were transformed with a series of *srp* deletion plasmids. The permissive temperature was reduced to 40 °C with selective pressure to maintain the plasmids. Transformed cells were examined for relative viability at 42 °C and 30 °C. Cells containing *p*HSG::*spc*, a vector plasmid, had a relative viability (RV) of less than 0.001. However, the RV was increased to 0.39 in cells containing *p*SRO1, which carried a 3.4 kb *srp/hsl* DNA fragment containing all five promoters (Fig. 1b).

We determined the effects of sequential gene deletions from the *srp/hsl* locus on *p*SRO1 on the complementation of the temperature sensitivity of AQ11591. Remarkably, the RV of the transformants carrying the *hslO* gene deletion (*p*SRO*ΔhslO*) was approximately 0.001. In contrast, introducing a 1.4 kb *hslR–hslO* DNA fragment (*p*hslO) rescued the temperature sensitivity of AQ11591 cells, indicating that the *hslO* gene is essential for activation of the Srp pathway. *p*SRO*Δ*L12 complemented the temperature sensitivity better than plasmids carrying an additional DNA fragment upstream of the *Afl*III restriction site (*p*SRO1). It seems likely that the increase in the RV resulted from the de-repression of *srp* genes caused by the deletion of the LexA boxes. We also examined the RVs of *p*SRO*Δ*L1 (a LexA box1 deletion derivative) and *p*SRO*Δ*P1 (a P1 promoter deletion derivative). The RVs were nearly identical in both strains and lower than those of the strain with *p*SRO*Δ*L12. Therefore, the P1 promoter had a small effect on complementation. Moreover, these results indicate that the expression of *hslO* is essential for the Srp pathway and, therefore, viability.

### *recAts polA* cells accumulated ROS at restrictive temperatures

**Fig. 2:**
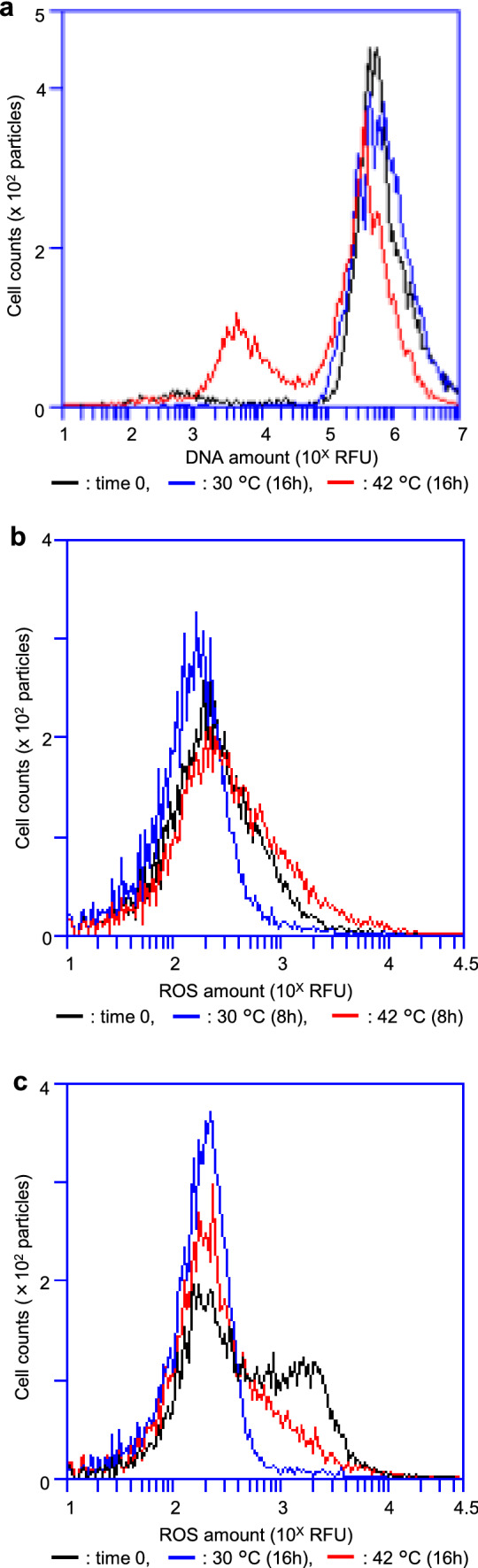
Comparison of the chromosome content and ROS accumulation in restrictive and permissive conditions for the *recAts polA* cells. **a** Comparison of DNA content for the *recAts polA* cells at permissive and restrictive temperatures. The DNA of the AQ10549 cells (*recAts polA*) was quantitatively compared as described in the Material and Methods section between the permissive and restrictive temperatures. Histograms show time 0 (black), 30 °C; 16 h (blue), and 42 °C; 16 h (red). A total of 10,000 particles are shown. **b** Comparison of ROS accumulation in restrictive and permissive conditions in *recAts polA* cells in a shift-up experiment. In the early logarithmic growth phase (OD_600_ = 0.1), the AQ10549 (*recAts polA*) culture was divided into two portions, and each was incubated at 30 °C and 42 °C. The ROS levels in the AQ10549 (*recAts polA*) cells were determined via Cell Rox Deep Red staining as described in the Materials and Methods section. The ROS levels were compared at the time of division (0) and 8 h after cultivation at 30 °C and 42 °C. Histograms show time 0 (black), 30 °C (blue), and 42 °C (red). A total of 10,000 particles are shown. **c** Comparison of ROS accumulation in restrictive and permissive conditions for the *recAts polA* cells during the inoculating experiment. AQ10549 (*recAts polA*) cells were inoculated with 1/100 of the overnight seed culture and incubated at 30 °C or 42 °C for 16 h. The ROS levels of the AQ10549 (*recAts polA*) cells were determined as shown in Fig. 2B, and the ROS levels of the seed and cultured cells at 30 °C or 42 °C for 16 h were compared. Histograms show time 0 (black), 30 °C (blue), and 42 °C (red). A total of 10,000 particles are shown

We next determined whether the chromosomes of *recA polA* cells were degraded at a restrictive temperature (42 °C). Temperature-sensitive AQ10549 cells were divided into two flasks in the early log phase and then cultivated at either 30 °C or 42 °C for 16 h. After cultivation for 16 h, the cells were stained with Pico Green for DNA quantification using FL-1A in our flow cytometer (Fig. 2a). As a single chromosome corresponded to approximately 10^5^ RFU (relative fluorescence units) in this assay, AQ10549 cells at time 0 (black) possessed both multi-nucleated particles at around 10^6^ RFU and cell debris at around 10^3^ RFU. Therefore, particles with less than a single chromosome (particles with < 5 × 10^4^ RFU) would be injured cells with degraded chromosomes (DC cells). The mean FL-1A, an average of DNA amounts, was 1.18 × 10^6^ RFU at time 0. After 16 h at 30 °C, AQ10549 cells yielded a peak at around 2 × 10^5^ RFU, corresponding to cells with a single chromosome. The mean FL-1A for the permissive condition at 16 h was 9.73 × 10^5^ RFU. A feature of AQ10549 cells under restrictive conditions for 16 h was that they possessed many anucleate cells between 10^3^ and 10^4^ RFU, suggesting that the cells had degrading chromosomes. The mean FL-1A for the restrictive condition was also 4.85 × 10^5^ RFU, i.e. a ratio of 0.75 compared to AQ10549 cells under permissive conditions (Fig. S4a). The ratio of DC cells to total particles was 0.3 at 42 °C, whereas it was 0.1 at 30 °C (Fig. S4b). Therefore, it is doubtful that these differences, both the average amounts of DNA and DC cell ratios, could explain the lethality.

This may explain why *recAts polA* cells failed to grow at a restricted temperature and how *hslO* recovered these cells. Recently, Hong et al. (2017, 2019) reported a relationship between lethality and ROS accumulation. We observed that a DsRed fluorescent protein that requires oxidation for maturation (Strack et al. 2010) occasionally failed to mature in the *ΔhslO* mutant cells. We hypothesised that the redox condition in *ΔhslO* cells was different from that of the wild-type cells; therefore, we attempted to analyse the ROS levels in *recA polA25* cells with a CellRox Deep Red using flow cytometry (McBee et al. 2017).

We assayed the effect of temperature shift-up in AQ10549 (*recA200 polA25*) cells on ROS with CellRox. At the log phase, half of the AQ10549 derivative cells were up-shifted to a restrictive temperature and compared with those of another portion at the permissive temperature (Fig. 2b). Histograms of the stained control AQ10549 cells were shifted to lower levels with further cultivation at 30 °C for 16 h (0 h: black, 16 h: blue). In contrast, there was a strongly stained population of AQ10549 cells at 42 °C for 16 h (red), suggesting that some AQ10549 populations responded to the temperature up-shift with increased ROS production. We compared the amount of ROS in the AQ10549 cells after inoculation with the AQ10549 strain at both 30 °C and 42 °C for 16 h (Fig. 2c). As a result, compared with AQ10549 cells used for inoculation (black), cells had lower ROS levels after 16 h of incubation at 30 °C than at inoculation (blue). Moreover, after 16 h of culturing cells at 42 °C, ROS levels were higher than those at 30 °C.

**Fig. 3:**
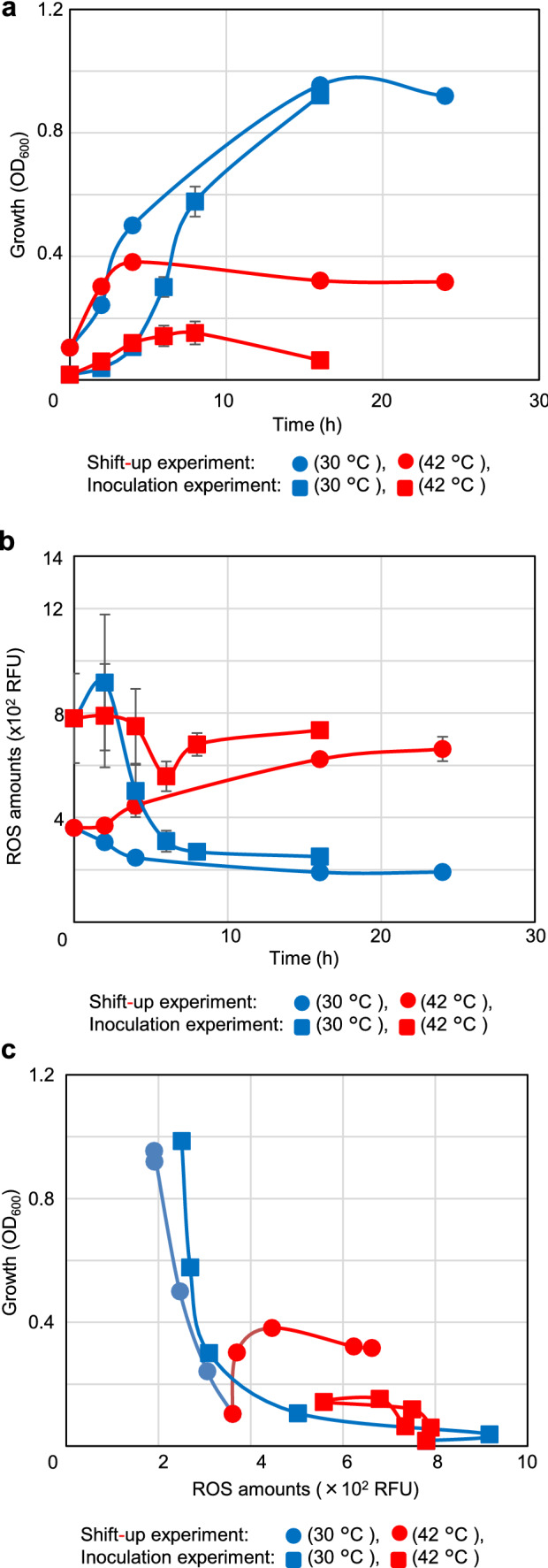
ROS accumulation in *recAts polA* cells in restricted conditions. **a** Inhibition of growth at a restricted temperature in the shift-up and inoculation experiments. The growth curves for the shift-up and inoculation experiments are shown. The results show the changes in absorbance (OD_600_) over time, with the shift-up experiment starting when the culture medium was divided. The inoculation experiment started at the time of inoculation. In the figure, the shift-up experiment is indicated using circles and the inoculation experiment using squares, at 30 °C (blue) and 42 °C (red), respectively. Each point is shown as standard error of the mean (n ≥ 3, SEM). **b** ROS accumulation at restricted temperatures for both the shift-up and inoculation experiments. For the shift-up and inoculation experiments shown in Fig. 3A, the mean ROS levels of the AQ10549 cells stained with CellRox DeepRed are shown over time as in (**a**). The shift-up experiments are identified using circles and the inoculation experiments using squares at 30 °C (blue) and 42 °C (red), respectively. Each point is shown as standard error of the mean (*n* ≥ 3, SEM). **c** Effects of temperature on both cell growth and the mean ROS during a time-course. For AQ10549 cell samples shown in **a** and **b**, two-dimensional plots were constructed with the mean ROS levels and absorbance on the x and y axes, respectively. Sequential measurement points were connected to better understand the changes. Shift-up experiments are indicated using circles and inoculation experiments using squares at 30 °C (blue) and 42 °C (red), respectively

Although the histograms shown in Fig. 2b and c present accurate information, they are difficult to understand due to pile up when many experimental conditions intended to compare. Therefore, we attempted to use the mean ROS levels for the cells. Cells were collected to determine their growth (Fig. 3a) and ROS levels (Fig. 3b) over time at both 30 °C and 42 °C. In the shift-up experiments, growth arrest was observed after 4 h of incubation at 42 °C (Fig. 3a). In contrast, it was observed after 8 h at 42 °C in the inoculation experiments. Interestingly, the mean ROS levels in the cells increased at or after those times in both the shift-up and inoculation experiments (Fig. 3b and c). In contrast, the mean ROS levels decreased after 2 h at 30 °C. To show the relationship between cell growth and ROS more comprehensively, the results above were interpolated along the X and Y axis for the mean ROS levels and growth, respectively, and the chronologically continuous measurement points were connected with lines (Fig. 3c). This result indicated that the mean ROS concentration in the cell population under restrictive conditions could be used to assess its effect on growth. Therefore, we attempted to investigate *recA polA* lethality using statistical analysis of the mean ROS levels.

**Fig. 4:**
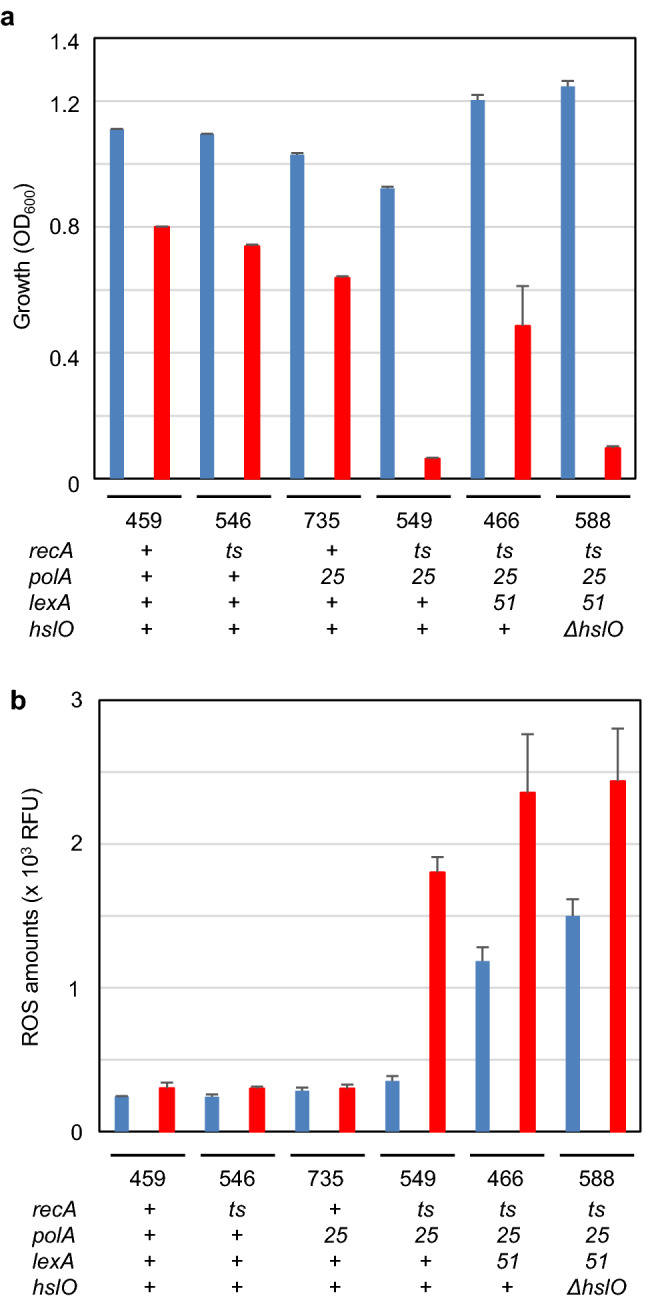
Genotype effects on growth and ROS accumulation in both permissive and restricted temperatures. **a** Genotype effects on growth at both permissive and restricted temperatures. Cells with individual genetic mutations were inoculated with 1/100 volumes of 2 mL of M9GCAA medium, and the absorbance values after 16 h of incubation were compared. Each bar represents standard error of the mean (*n* ≥ 3, SEM); 30 °C (blue) and 42 °C (red). Below, 3 figure numbers are corresponding to their last 3 figure number of the strain. The genotypes were as follows: 459: AQ 10,459 (*recA200 lexA51*), 546: AQ10546 (*recA200*), 735: AQ11735 (*polA25*), 549: AQ10549 (*recA200 polA25*) 466: AQ11466 (*recA200 polA25 lexA51*) 588: AQ11588 (*recA200 polA25 lexA51 ΔhslO*). **b** Genotype effects on ROS accumulation in both permissive and restricted temperatures. Cells with individual genetic backgrounds were subjected to inoculation experiments, as shown in **a**, to compare the mean ROS levels after 16 h. The experimental conditions and cells were the same as those in **a**. Each bar represents standard error of the mean (*n* ≥ 3, SEM); 30 °C (blue) and 42 °C (red)

We examined whether *recA polA* lethality was synchronised with ROS accumulation in terms of genotype. *recA polA* lethality was a synthetic lethality consisting of two mutations that were not lethal in a single mutation. Therefore, we compared mean ROS levels in inoculation experiments after 16 h at both 30 °C and 42 °C on the wild, *polA, recAts, recAts polA* double mutant, and *recAts polA lexA51* (lethality suppressed cells AQ11466) and its *hslO* deletion derivative (Fig. 4a and b). As a result, each mutation alone was viable, whereas the *recA polA* double mutant cells (AQ10549) and suppressed cells with *ΔhslO* mutation (AQ11588) failed to grow at 42 °C (Fig. 4a). Therefore, we analysed the mean ROS levels in these strains. As a result, the mean ROS levels of the double mutant cells (AQ10549) at 42 °C were fivefold higher than those of the other single mutant cells (Fig. 4b). In the suppressed cells (AQ11466), the mean ROS levels increased even at 30 °C, contrary to our expectations. These results suggested that the growth inhibition and ROS accumulation were inversely correlated among the *lexA*^+^ non-suppressed cells, whereas suppressed cells with the *lexA* mutation could grow even when the mean ROS levels were too high.

### Suppressed cells became resistant to the ROS-inducing reagent menadione, and *hslO* was involved in menadione tolerance

We confirmed that temperature sensitivities were synchronised with ROS accumulation among *lexA*^+^ cells, and determined whether ROS induction by menadione could be related to growth inhibition among non-suppressed and suppressed cells at 30 °C. Menadione, a vitamin K derivative, exogenously induces ROS, mainly consisting of superoxide (Mori et al. 2008). It was used to study the effects of ROS on cell growth. Menadione has been used previously as a model compound in oxidative stress research (Criddle et al. 2006; Tani et al. 2007).

**Fig. 5:**
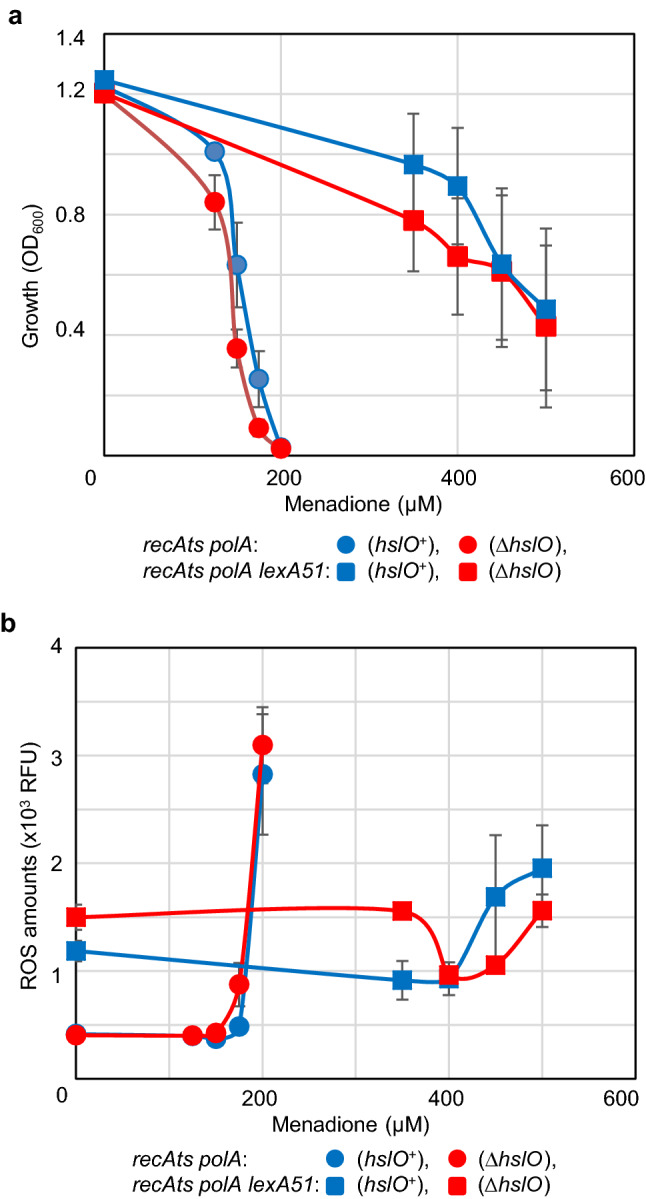
Menadione effects on cell growth and ROS accumulation in *recAts polA* cells. **a** Inhibition of *recAts polA* cell growth with menadione additions to the medium. The effects of menadione on the growth of the *recAts polA* cells were compared using inoculation experiments. Each determination was measured 16 h post inoculation. The *recAts polA* cells are indicated using circles, with the *hslO* wild-type strain (TK10549) in blue and the *hslO* mutant strain (TK3276) in red. *recAts polA lexA*(Def) strain is indicated using squares, with the *hslO* wild-type strain (TK11466) in blue and the *hslO* mutant strain (TK11588) in red. Each point is shown as standard error of the mean (*n* ≥ 3, SEM). **b** ROS accumulation with menadione additions to the medium in *recAts polA* cells. The effects of menadione on the accumulation of ROS in *recAts polA* cells were compared using inoculation experiments. Each determination was measured 16 h after the inoculation. The line graphs are shown as in **a**. Each point is shown as standard error of the mean (*n* ≥ 3, SEM)

ROS accumulation and growth inhibition were observed after 16 h of culture at 42 °C (Figs. 3 and 4). Therefore, cells inoculated with the indicated menadione concentrations were analysed for growth (Fig. 5a) and mean ROS levels (Fig. 5b) after 16 h at 30 °C. As a result, the growth of non-suppressed *lexA*^+^ cells (AQ10549 and its *hslO* deletion strain TK3276) was remarkably inhibited when the menadione concentration was above 175 μM (Fig. 5a). In contrast to the non-suppressed cells, the suppressed *lexA51* cells (AQ11466 and its *hslO* deletion strain AQ11588) were substantially tolerant to menadione at 30 °C. Among the non-suppressed cells (AQ10549 and TK3276), the *ΔhslO*-deleted cells (TK3276) were slightly sensitive to menadione in terms of growth. This is consistent with studies on hydrogen peroxide sensitivity in *hslO* mutant cells (Jakob et al. 1999), as ROS can be metabolically converted to hydrogen peroxide. Therefore, the mean ROS levels of non-suppressed cells (AQ10549 and TK3276) were analysed. These results were in good agreement with their growth. The mean ROS levels drastically increased from menadione 175 μM (Fig. 5b). In addition, the suppressed cells (AQ11466 and AQ11588) were remarkably more tolerant than the non-suppressed cells in terms of ROS induction by menadione. These results suggested that the suppressed cells with the *lexA* mutation grew even with increased intracellular ROS levels. These results also showed synchrony in the mean ROS levels and growth inhibition in the exogenous ROS induction experiment.

**Fig. 6:**
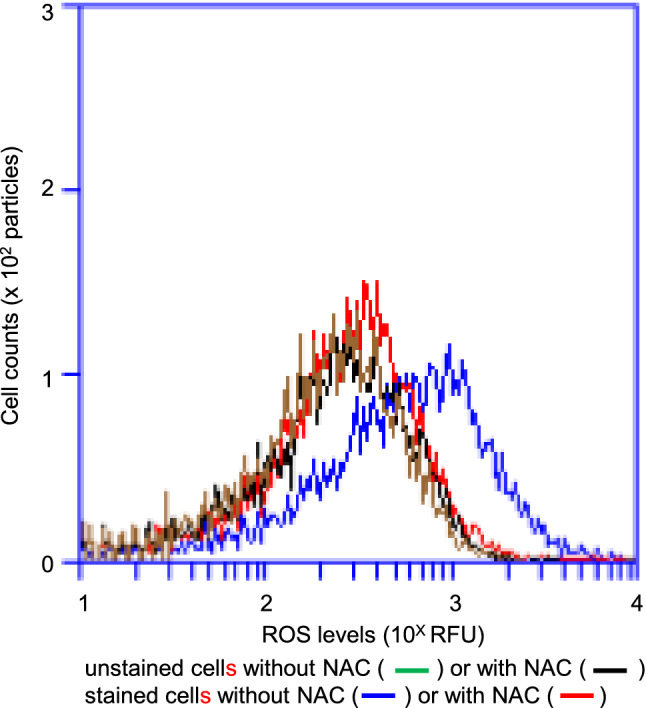
Decreasing ROS levels with N-acetyl cysteine visualised using CellRox staining of *recAts polA* cells. AQ10549 cells were cultured at 30 °C until the late logarithmic growth phase and divided into two equal volumes. A final concentration of 2.5 µM N-acetyl cysteine (NAC) was added to one of the flasks, and both flasks were then incubated at 30 °C for 1 h, following which ROS levels were measured as per the experimental procedure. The ROS levels and cell numbers are shown as FL-4A (*x* axis) and cell numbers (*y* axis). The cells represented by the colours in the histograms are as follows: For cells without NAC, Cell Rox unstained cells are shown in green and stained cells, in blue; for cells with NAC, CellRox unstained cells are shown in black and stained cells, in red. A total of 10,000 particles are shown

The difference between the non-suppressed and suppressed cells was likely associated with the *lexA* mutation. *hslO* was seemingly weakly induced by SOS induction (Fig. S3). Therefore, we further investigated whether the presence or absence of an *hslO* plasmid affected the growth and the mean ROS levels upon addition of menadione (Fig. S5a and b). Both non-suppressed (AQ10549) and suppressed cells with *ΔhslO* mutation (AQ11588 quadruple mutant) were transformed with either the vector or a *hslO* expression plasmid (*p*EXsrpC). The suppressed cells with the *ΔhslO* mutation (AQ11588) harboring the *hslO* expression plasmid (TK4559) were less affected by the addition of menadione than the corresponding vector cells (TK4558), resulting in the suppression of menadione-induced growth inhibition and ROS accumulation (Fig. S5ab). In order to validate our experimental system, we investigated whether ROS could be detected with CellRox with or without 2.5 mM N-acetyl cysteine (NAC) (De la Fuente and Victor 2001), a radical scavenger. AQ10549 cells were divided into two flasks at the late log phase, after which NAC was added to one of the flasks. After cultivation for 1 h at 30 °C, the cells were stained using CellRox, which was detected with FL-4A in our flow cytometer (Fig. 6). Untreated AQ10549 cells were stained using CellRox, whereas cells treated with NAC were not, indicating ROS scavenger sensitivity. Thus, it was confirmed that ROS-producing cells could be detected using this experimental procedure. Therefore, the suppression of temperature sensitivity in *recAts polA* cells seemed to be closely related to the intracellular ROS levels, both in terms of the *lexA* and *hslO* requirements.

**Fig. 7:**
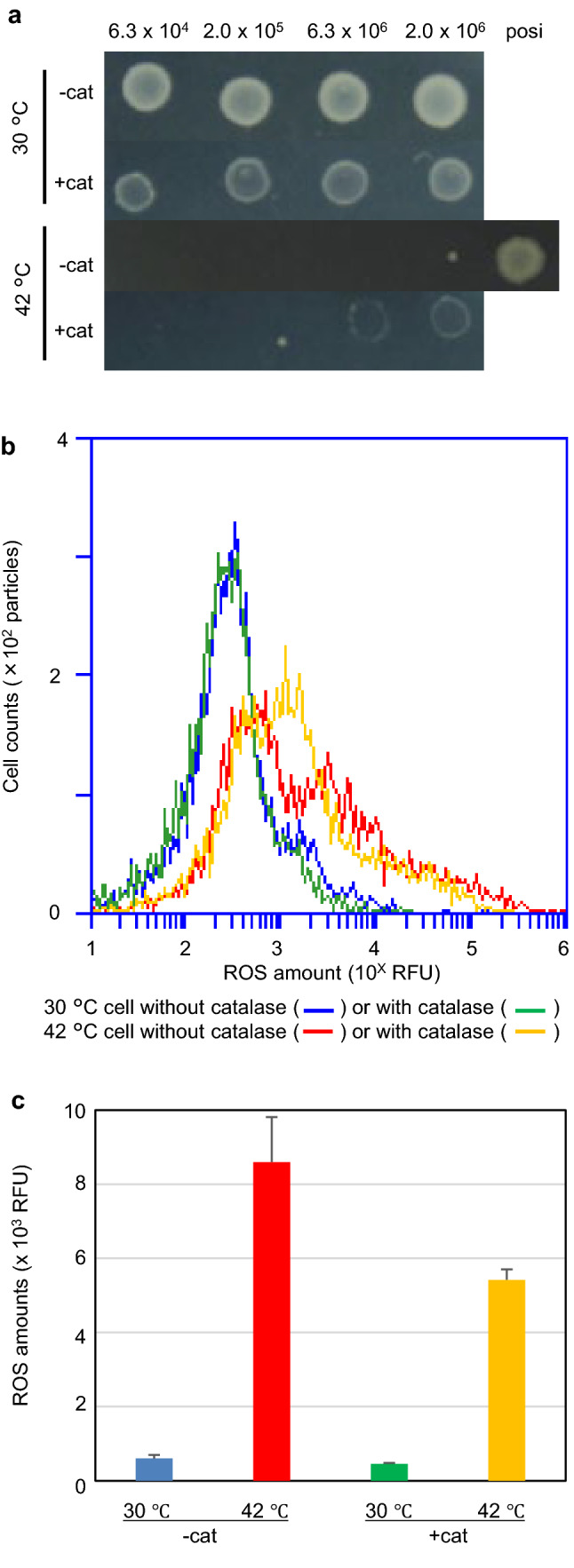
Effects of catalase additions to the plate medium on colony formation and ROS accumulation in *recAts polA* cells. **a** Effects of catalase additions to the plate medium on the colony formation of *recAts polA* cells. *recAts polA* (AQ10549) cells were spotted with the indicated number of particles and incubated at 30 °C and 42 °C. The number of particles between each spot ranged from 6.3 × 10^4^ to 2 × 10^6^ cells and varied √tenfold at each dilution. Posi indicates 6.3 × 10^4^ cells of TK10458. A combination of the photos was shown from top to bottom, with a combination of no catalase and 1000 U/mL, both at 30 °C and 42 °C, respectively. **b** Effects of catalase additions to the plate medium on ROS accumulation of *recAts polA* cells. The cells shown in (**a**) were recovered and stained with SYBR Green I and CellRox DeepRed. Nucleic acid-containing *Escherichia coli* particles were selected from the double-stained samples, as described in the Materials and Methods, and were analysed using histograms. Each histogram was as follows: blue, 30 °C without catalase; green, 30 °C with catalase; red, 42 °C without catalase; orange, 42 °C with catalase. **c** Effects of catalase additions to the plate medium on ROS accumulation in *recAts polA* cells with mean ROS levels. Cells were recovered as shown in **b**, stained with SYBR Green I and CellRox DeepRed. The mean ROS levels for the *E. coli* particles with nucleotides were determined as described in the “Materials and Methods”. The colour key for the bars is shown in **b**. Each bar represents standard error of the mean (*n* ≥ 3, SEM)

The aforementioned experimental results indicated that intracellular ROS levels played an important role in the growth inhibition of *recAts polA* cells and suppression of lethality by inducing SOS expression. ROS produced by respiration and biological reactions are converted into various active molecular species via intracellular metabolism (Iuchi and Weiner 1996; Vaze et al. 2017). One of them is hydrogen peroxide, which is converted into more dangerous hydroxyl radicals. In contrast, bipyridine and NAC are ROS scavengers. However, these reagents also affect the essential metabolic reactions in cells. Therefore, we used catalase to study the effects of hydrogen peroxide degradation. The AQ10549 (*recAts polA*) cells grew well after 16 h at permissive temperature (30 °C) but failed to grow on a normal M9GCAA agar plate at the restrictive temperature (42 °C). However, when the M9GCAA agar plate was supplemented with catalase at a final concentration of 1,000 U/mL (+ cat), AQ10549 cells showed growth in the presence of both 2 × 10^6^ and 6.33 × 10^5^ cells (Fig. 7a). Therefore, *E. coli* spotted on the agar plates were collected by tips, and the ROS levels of the bacterial particles were measured using flow cytometry. To eliminate the influence of agar particles, bacterial particles were double-stained with SYBR Green I and CellRox DeepRed. The ROS levels for the particles with nucleic acids were compared. We could not detect any accumulation of ROS at 30 °C with or without (blue) catalase (Fig. 7b). The resulting histograms were almost identical. As expected, cells from 42 °C without catalase showed increased ROS levels. In contrast, ROS levels in cells from a catalase-containing medium at 42 °C were reduced compared to those from cells without catalase. The mean intracellular ROS levels were also analysed. These results showed that the mean ROS levels from the catalase-containing medium at 42 °C were lower than those from the catalase-free medium (Fig. 7c). These results were consistent with a decrease in ROS in the histogram and indicate that the temperature sensitivity of *recAts polA* cells was partly owing to ROS caused by hydrogen peroxide and its metabolites. Thus, ROS may regulate cell growth, presumably via ROS-sensitive cell metabolism.

## Discussion

In this study, we investigated what occurred in r*ecAts polA* cells with the Srp pathway, which suppresses lethality in *recA polA* cells, and showed that the temperature sensitivity of *recAts polA* cells was bacteriostatic rather than bactericidal. This indicates that there is a linkage between chromosome damage, intracellular ROS levels, and bacterial growth, as discussed later.

Lethal treatments, including antibiotics (Kohanski et al. 2010), trigger self-amplifying ROS accumulation (Hong et al. 2019). Thus, primal chromosome breakage would induce ROS production (Duquette et al. 2018), resulting in ROS self-amplification. This could result in growth retardation of *recAts polA* cells. It is currently unknown how DNA damage generates initial ROS in *recAts polA* cells. However, *E. coli* cells have been reported to produce a hydroxyl radical after hydroxyurea (HU) treatment (Bollenbach and Kishony 2009), resulting in the alteration of cytochrome oxidases (Davies et al. 2009). It is of interest that both HU stress and *recAts polA* lethality are incidents that occur at the replication fork. Simmons et al. (2008) mentioned the significance of the SOS response in coordinating DNA damage recognition and DNA replication. This might be related to the repression of *recAts polA* lethality by *lexA*(Def). It was also reported that detectable ROS are generated in response to DNA damage by chromatin remodelling factors (Duquette et al. 2018). Thus, it is possible that some replication forks (Nazaretyan et al. 2018) and/or nucleoid-associated factors sense DNA damage, and this triggers primal ROS production.

Interestingly, DNA checkpoints can discontinue the cell cycle to provide cells with time to deal with DNA damage (Parrilla-Castellar et al. 2004). The *hslO* mutation sensitises cells to hydrogen peroxide (Jakob et al. 1999) and fails to respond to hydrogen peroxide at elevated temperatures (Ilbert et al. 2007). The *hslO* expression rescues cells when they produce detrimental levels of ROS. ROS production is regulated by cell metabolism and reducing reagents, such as catalase. Although hydrogen peroxide and superoxide anions do not oxidise DNA directly, they feed directly or indirectly into the generation of the highly reactive hydroxyl radical that damages the bacterial chromosome (Mendoza-Chamizo et al. 2018). Thus, a reduction in the initial ROS concentration will effectively reduce damage or provide response time for the required repairs. Interestingly, redox-sensitive alterations of replisome have been reported to act as safeguards for the genome (Somyajit et al. 2017). The slowdown in replisome activity is a strategy used to prevent clashes with engaged DNA repair proteins and preserve the integrity of the replication fork (Soubry et al. 2019).

An ROS-mediated lethal mechanism (Kohanski et al. 2007) and a redox-signalling pathway (Sporer et al. 2017) have been reported in *E. coli*. As shown in this study, relatively high ROS levels result in growth arrest. It was noteworthy that up to two cleavage sites on a single chromosome could cause growth arrest owing to the self-amplification process of ROS produced in the early stages. Conversely, high ROS levels causing this arrest would be reduced to acceptable levels by redox molecular chaperones such as HslO, possibly collaborating with catalase and superoxide dismutase. It was also possible to orchestrate cellular responses to high levels of ROS, such as the SOS response under the regulation of *lexA,* as shown in this study. In regard to transcription, hydrogen peroxide binds to OxyR (Pedre et al. 2018), an oxidative stress transcription factor, evoking an oxidative stress response. Given that ROS themselves are highly reactive inhibitory effectors, they might participate in a series of signal amplification, signal elimination, tuning, memory systems, and transcription using ROS themselves as the effectors and signal transducers. In this case, we hypothesised that a signal transduction mechanism consisting of ROS and their metabolites might govern DNA damage repair. Cellular responses to ROS would not preclude this possibility. Thus, the redox-signalling pathway might be a pivotal mechanism underlying and regulating DNA metabolism in *E. coli*.

Using a synthetic lethality experimental model of chromosome damage, we found that growth arrest of *recAts polA* cells was synchronised with elevated intracellular ROS levels at restricted temperatures. This growth arrest was ameliorated by the elimination of hydrogen peroxide, indicating that *recAts polA* lethality is bacteriostatic and mediated at least in part by ROS. Importantly, these studies provide new insights into the relationship between DNA damage and redox signalling. Further research on such interactions is pivotal for understanding how DNA damage signal transduction regulates cell proliferation. Furthermore, the question of how *recAts polA* cells cope with chromosome breakage remains. RecA-independent conjugal recombination has recently been reported (Kingston et al. 2015, 2017). ROS-induced BRCA1/2-independent homologous recombination has also been reported (Teng et al. 2018). The continued study of damage response mechanisms such as replication fork restart in damaged cells will help further elucidate how chromosomal integrity is maintained.

## Supplementary Information

Below is the link to the electronic supplementary material.

Supplementary file1 (PDF 475 KB)

## Data Availability

The data that support the findings of this study are available from the corresponding author upon reasonable request.

## References

[CR1] Asai T, Sommer S, Bailone A, Kogoma T (1993). Homologous recombination-dependent initiation of DNA replication from DNA damage-inducible origins in *Escherichia coli*. EMBO J.

[CR2] Asai T, Bates DB, Kogoma T (1994). DNA replication triggered by double-stranded breaks in *E. coli*: dependence on homologous recombination functions. Cell.

[CR3] Blattner FR (1997). The complete genome sequence of *Escherichia coli* K-12. Science.

[CR4] Bollenbach T, Kishony R (2009). Hydroxyurea triggers cellular responses that actively cause bacterial cell death. Mol Cell.

[CR5] Cao Y, Kogoma T (1995). The mechanism of recA polA lethality: suppression by RecA-independent recombination repair activated by the lexA(Def) mutation in *Escherichia coli*. Genetics.

[CR6] Cao Y, Rowland RR, Kogoma T (1993). DNA polymerase I and the bypassing of RecA dependence of constitutive stable DNA replication in *Escherichia coli* rnhA mutants. J Bacteriol.

[CR7] Criddle DN (2006). Menadione-induced reactive oxygen species generation via redox cycling promotes apoptosis of murine pancreatic acinar cells. J Biol Chem.

[CR8] Davies BW, Kohanski MA, Simmons LA, Winkler JA, Collins JJ, Walker GC (2009). Hydroxyurea induces hydroxyl radical-mediated cell death in *Escherichia coli*. Mol Cell.

[CR9] De la Fuente M, Victor VM (2001). Ascorbic acid and *N*-acetylcysteine improve in vitro the function of lymphocytes from mice with endotoxin-induced oxidative stress. Free Radic Res.

[CR10] Decros G (2019). Get the balance right: ROS Homeostasis and redox signalling in fruit. Front Plant Sci.

[CR11] Duquette ML, Kim J, Shi LZ, Berns MW (2018). LSD1 mediated changes in the local redox environment during the DNA damage response. PLoS ONE.

[CR12] Ferullo DJ, Cooper DL, Moore HR, Lovett ST (2009). Cell cycle synchronization of *Escherichia coli* using the stringent response, with fluorescence labeling assays for DNA content and replication. Methods.

[CR13] Hong Y, Li L, Luan G, Drlica K, Zhao X (2017). Contribution of reactive oxygen species to thymineless death in *Escherichia coli*. Nat Microbiol.

[CR14] Hong Y, Zeng J, Wang X, Drlica K, Zhao X (2019). Post-stress bacterial cell death mediated by reactive oxygen species. Proc Natl Acad Sci USA.

[CR15] Ilbert M (2007). The redox-switch domain of Hsp33 functions as dual stress sensor. Nat Struct Mol Biol.

[CR16] Imlay JA (2015). Diagnosing oxidative stress in bacteria: not as easy as you might think. Curr Opin Microbiol.

[CR17] Iuchi S, Weiner L (1996). Cellular and molecular physiology of *Escherichia coli* in the adaptation to aerobic environments. J Biochem.

[CR18] Jakob U, Muse W, Eser M, Bardwell JC (1999). Chaperone activity with a redox switch. Cell.

[CR19] Karp PD (2018). The EcoCyc database. EcoSal plus.

[CR20] Kingston AW, Roussel-Rossin C, Dupont C, Raleigh EA (2015). Novel recA-independent horizontal gene transfer in Escherichia coli K-12. PLoS ONE.

[CR21] Kingston AW, Ponkratz C, Raleigh EA (2017). Rpn (YhgA-like) proteins of Escherichia coli K-12 and their contribution to RecA-independent horizontal transfer. J Bacteriol.

[CR22] Kleckner N, Bender J, Gottesman S (1991). Uses of transposons with emphasis on Tn10. Methods Enzymol.

[CR23] Kogoma T (1997). Stable DNA replication: interplay between DNA replication, homologous recombination, and transcription. Microbiol Mol Biol Rev.

[CR24] Kohanski MA, Dwyer DJ, Hayete B, Lawrence CA, Collins JJ (2007). A common mechanism of cellular death induced by bactericidal antibiotics. Cell.

[CR25] Kohanski MA, Dwyer DJ, Collins JJ (2010). How antibiotics kill bacteria: from targets to networks. Nat Rev Microbiol.

[CR26] Korber P, Stahl JM, Nierhaus KH, Bardwell JC (2000). Hsp15: a ribosome-associated heat shock protein. EMBO J.

[CR27] Kowalczykowski SC, Dixon DA, Eggleston AK, Lauder SD, Rehrauer WM (1994). Biochemistry of homologous recombination in *Escherichia coli*. Microbiol Rev.

[CR28] Kuznetsova E (2006). Genome-wide analysis of substrate specificities of the *Escherichia coli* haloacid dehalogenase-like phosphatase family. J Biol Chem.

[CR29] Lennox ES (1955). Transduction of linked genetic characters of the host by bacteriophage P1. Virology.

[CR30] Manoil D, Bouillaguet S (2018). Oxidative stress in bacteria measured by flow cytometry. Adv Biotechnol Microbiol.

[CR31] McBee ME, Chionh YH, Sharaf ML, Ho P, Cai MW, Dedon PC (2017). Production of superoxide in bacteria is stress- and cell state-dependent: a gating-optimized flow cytometry method that minimizes ROS measurement artifacts with fluorescent dyes. Front Microbiol.

[CR32] Mendoza-Chamizo B, Løbner-Olesen A, Charbon G (2018). Coping with reactive oxygen species to ensure genome stability in *Escherichia coli*. Genes.

[CR33] Miller JH (1992). A short course in bacterial genetics: a laboratory manual and handbook for Escherichia coli and related bacteria.

[CR34] Monk M, Kinross J (1972). Conditional lethality of recA and recB derivatives of a strain of E*scherichia coli* K-12 with a temperature-sensitive deoxyribonucleic acid polymerase I. J Bacteriol.

[CR35] Mori T, Suenaga H, Miyazaki K (2008). A metagenomic approach to the identification of UDP-glucose 4-epimerase as a menadione resistance protein. Biosci Biotechnol Biochem.

[CR36] Nazaretyan SA, Savic N, Sadek M, Hackert BJ, Courcelle J, Courcelle CT (2018). Replication rapidly recovers and continues in the presence of hydroxyurea in *Escherichia coli*. J Bacteriol.

[CR37] Nikitaki Z, Hellweg CE, Georgakilas AG, Ravanat JL (2015). Stress-induced DNA damage biomarkers: applications and limitations. Front Chem.

[CR38] Parrilla-Castellar ER, Arlander SJ, Karnitz L (2004). Dial *9–1-1* For DNA damage: the Rad9-Hus1-Rad1 (9–1-1) clamp complex *Dial*. DNA Repair.

[CR39] Pedre B (2018). Structural snapshots of OxyR reveal the peroxidatic mechanism of H_2_O_2_ sensing. Proc Natl Acad Sci USA.

[CR40] Prentki P, Krisch HM (1984). In vitro insertional mutagenesis with a selectable DNA fragment. Gene.

[CR41] Reichmann D (2012). Order out of disorder: working cycle of an intrinsically unfolded chaperone. Cell.

[CR42] Sambrook J, Fritsch EF, Maniatis T (1989). Molecular cloning: a laboratory manual.

[CR43] Simmons LA, Foti JJ, Cohen SE, Walker GC (2008). The SOS regulatory network. EcoSal plus.

[CR44] Somyajit K (2017). Redox-sensitive alteration of replisome architecture safeguards genome integrity. Science.

[CR45] Soubry N, Wang A, Reyes-Lamothe R (2019). Replisome activity slowdown after exposure to ultraviolet light in *Escherichia coli*. Proc Natl Acad Sci USA.

[CR46] Sporer AJ, Kahl LJ, Price-Whelan A, Dietrich LEP (2017). Redox-based regulation of bacterial development and behavior. Annu Rev Biochem.

[CR47] Strack RL, Strongin DE, Mets L, Glick BS, Keenan RJ (2010). Chromophore formation in DsRed occurs by a branched pathway. J Am Chem Soc.

[CR48] Suzuki S, Kaidow A, Meya T, Masuya A, Shiina T (2017). Phenotypic difference between Δ(srl-recA)306 and ΔrecA: Km elucidated by next-generation sequencing combined with a long-PCR system. J Gen Appl Microbiol.

[CR49] Tani S, Yonezawa Y, Morisawa S, Nishioka H (2007). Development of a new *E. coli* strain to detect oxidative mutation and its application to the fungicide o-phenylphenol and its metabolites. Mutat Res.

[CR50] Teng Y (2018). ROS-induced R loops trigger a transcription-coupled but BRCA1/2-independent homologous recombination pathway through CSB. Nat Commun.

[CR51] Vaze ND, Park S, Brooks AD, Fridman A, Joshi SG (2017). Involvement of multiple stressors induced by non-thermal plasma-charged aerosols during inactivation of airborne bacteria. PLoS ONE.

[CR52] Wade JT (2006). Extensive functional overlap between sigma factors in *Escherichia coli*. Nat Struct Mol Biol.

[CR53] Walker GC (1996). Escherichia coli and Salmonella thyphimurum: cellular and molecular biology.

[CR54] Wilson K (2001). Preparation of genomic DNA from bacteria. Curr Protoc Mol Biol.

[CR55] Zeman MK, Cimprich KA (2014). Causes and consequences of replication stress. Nat Cell Biol.

