# Supplementary material for: Reactive oxygen species accumulation is synchronised with growth inhibition of temperature-sensitive recAts polA Escherichia coli
Source: Arch Microbiol. 2022 Jun 16;204(7):396. doi: 10.1007/s00203-022-02957-z (PMC9200703; doi:10.1007/s00203-022-02957-z)
Supplement: Supplementary file 1 — Supplementary file1 (PDF 475 KB) [file 203_2022_2957_MOESM1_ESM.pdf]

Supplementary Information

Reactive oxygen species accumulation is synchronised with growth inhibition of temperature-sensitive *recAts polA Escherichia coli*

Journal name: **Archives of Microbiology**

Akihio Kaidow*[1], Noriko Ishii[1], Sinngo Suzuki[2], Takashi Shiina[2], and Hirokazu Kasahara[1]

[1]Department of Bioscience and Technology, School of Biology, Tokai University, Sapporo 005-8601, Japan
[2]Department of Molecular Medicine, School of Medicine, Tokai University, Isehara 259-1193, Japan

[*]Corresponding author: e-mail: akaidow@tsc.u-tokai.ac.jp; phone: 81-11- 571-5111 (ex2912); fax: 81-11-571-

Supplementary Methods

β-galactosidase assay of *srp* promotors

To construct reporter plasmids, the DNA fragments were cloned into pRS415. These included the P1 promoter containing *Stu*I-*Nsi*I DNA fragment just upstream of the *yrfG* gene (pAQ11246). To construct AQ11120 and AQ11122, P1 lysate from AQ8300 (AQ634 *malF3089*::Tn*10 lexA3*) was transduced into JM103. Tcr colonies were selected, and UV sensitivities were confirmed. UV-resistant and sensitive colonies were designated as AQ11120 (*lexA*$^+$) and AQ11122 (*lexA3*), respectively. Those derivatives were grown on LA plates at 37 °C. Exposure to UV light was carried out at a cell density of 1 x 10^8 cells/mL. The β-galactosidase activity was determined as described by Miller (Miller 1992).

Supplementary Figures

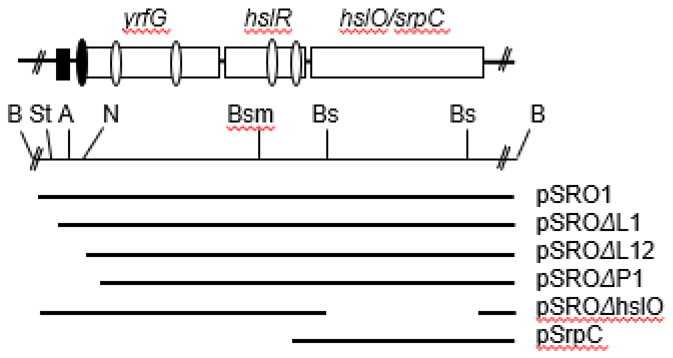

Fig. S1. Gene organisation of *hsl* locus and *srp* plasmids
Gene organisation of the *hsl* locus. The gene organisation of this region is shown. The putative *srp* operon genes are indicated with open boxes. Black boxes upstream of the *yrfG* indicate LexA-like sequences. The arrowhead indicates the position of the miniTn*10* insertion in the *srp-529* mutation. Double slash indicates omission of chromosome region. Promoters in the *srp* region are shown as ovals: P1 promoter (black oval). The restriction endonuclease cleavage sites are identified as follows: B; *Bam*HI, St; *Stu*I, A; *Afl*III, N; *Nsi*I, Bsm; *Bsm*I and Bs; *Bst*EII.

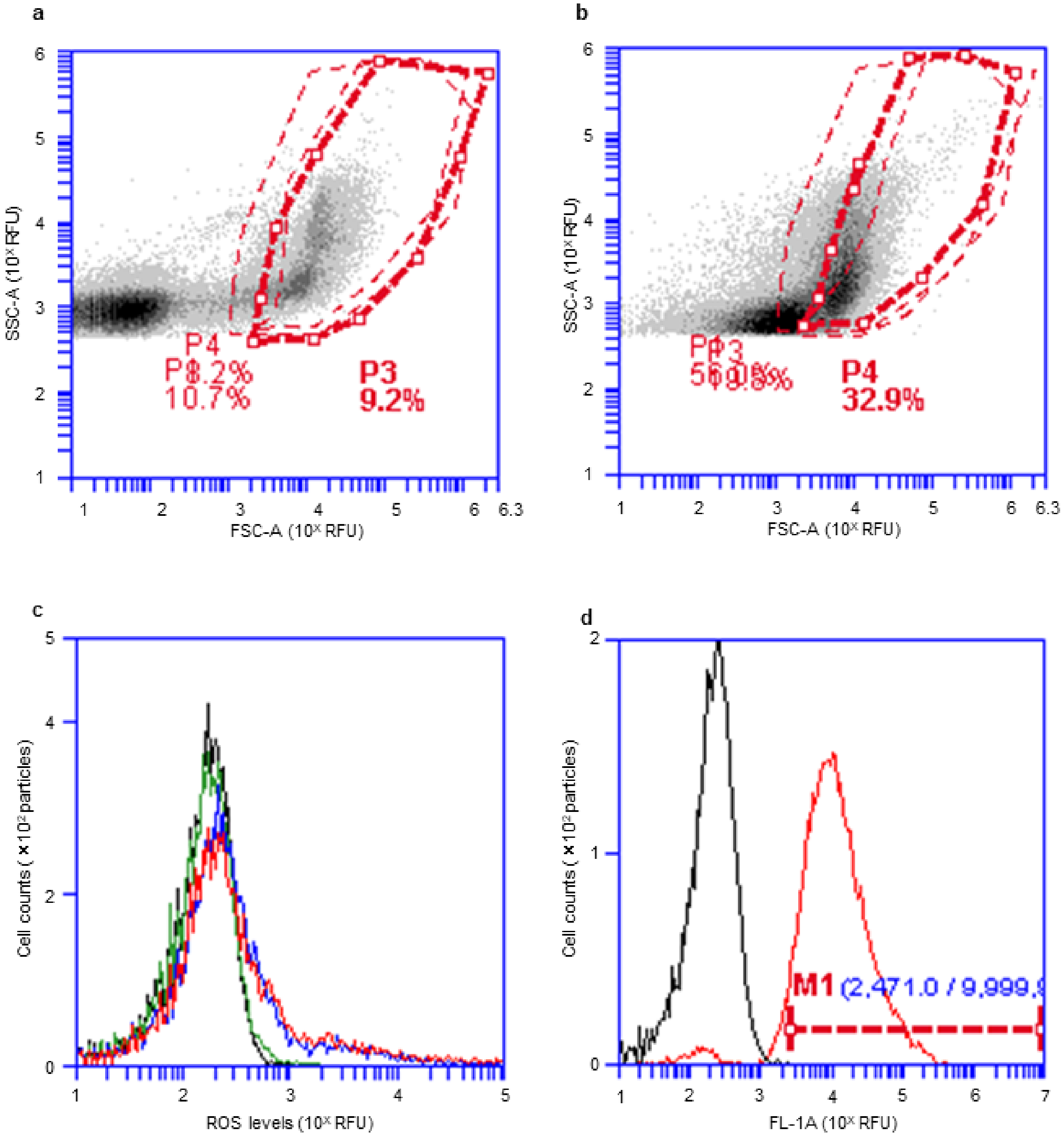

Fig. S2. Setting gates, P3, P4, and M1, for cell particle analysis

(a) P3 used for the analysis of cell particles. The P3 gate was used to distinguish bacterial particles from cell debris during quantitative chromosome analysis and ROS measurements. The P3 section showed bacterial particles, and the set in the lower left part outside the P3 section showed cell debris and microparticles in the culture medium. The x-axis represents the FSC-A: forward scatter, which is the size of the cell, and the y-axis represents the SSC-A: side scatter, which is the complexity of the internal structure of the cell. AQ10549 cells were shown to have 50,000 particles in the P3 compartment after 4 h of incubation at the restrictive temperature using a shift-up

experiment. (**b**) P4 used for the analysis of cell particles. The P4 gate was used for qualitative analysis of the chromosomes and ROS measurements of the bacterial particles grown on the agar plate are shown with recovered particles. The P4 gate was set to be narrower than the P3 gate to reduce the effects of the agar-derived particles. (**c**) Effect of SYBR green I staining on the detection of ROS signals in the FL-4A channel. To analyse the effect of SYBR Green I staining on ROS detection, we compared no staining (black), Syber green I single staining (blue), and Syber green I and Cellrox DeepRed double staining (red). The particles represented 10,000 particles in the P4 gate. (**d**) M1 gate used for selection of the particles with nucleotides. The histograms of unstained particles (black) and Syber Green I-stained particles (red) obtained from 10,000 particles in the P4 gate are shown. The M1 gate was set to possess less than 0.1% unstained particles in the gate to analyse particles with nucleic acids.

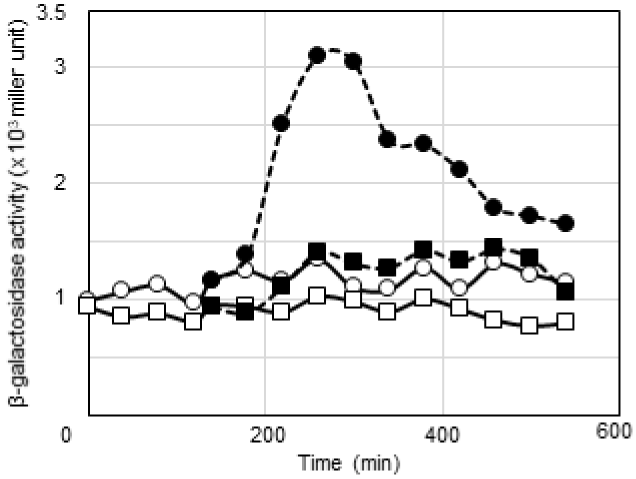

Fig. S3. Damage inducibility and effects of *lexA* (ind-) on *srp* promoters.

After cell densities reached 1 × 10^8 cell/mL (140 min) at 37 °C, logarithmically growing cultures were divided into two aliquots, and after exposure for 140 min to UV light (5 J/cm^2) or unirradiated conditions, β-galactosidase production in the uninduced (open symbols) and induced (closed symbols) cultures was measured as previously descried (Miller 1992) at the indicated times. AQ1851 (JM103) derivatives, AQ11120 (*lexA*$^+$) cells, and AQ11122 (*lexA3*) cells, were transformed using the P1 promoter (black oval) reporter plasmid, pAQ11246. The β-galactosidase activities of the reporter plasmid harbouring AQ11120 (*lexA*$^+$: circle) cells and AQ11122 (*lexA3:* square) are shown.

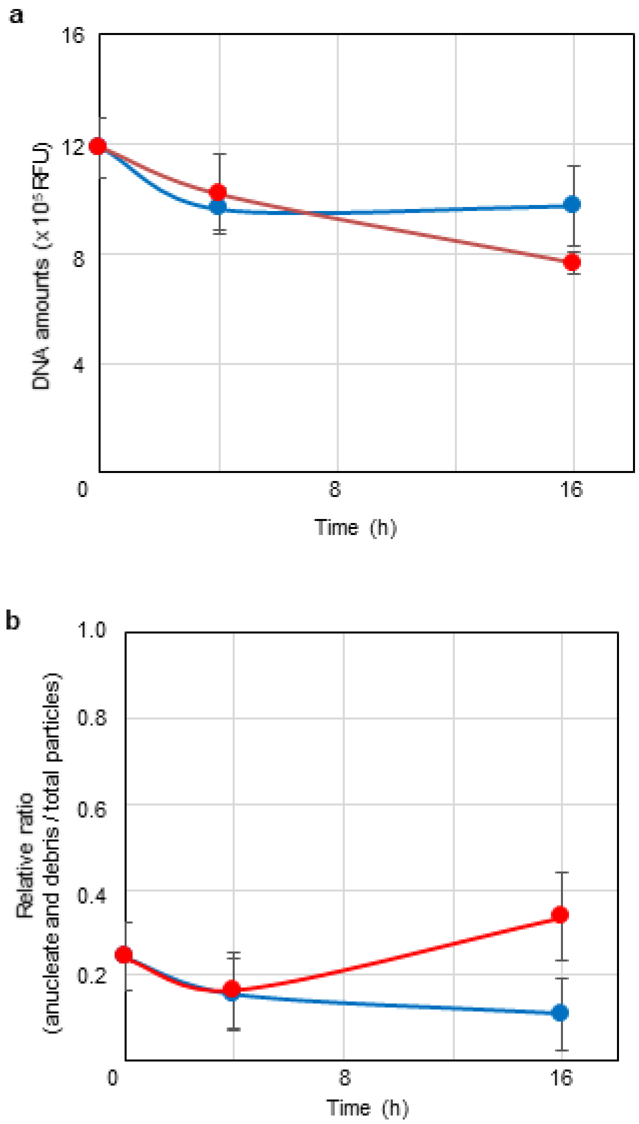

Fig. S4. Effects of restricted temperatures on the quantity of DNA and anucleate cell production in *recAts polA* cells

(**a**) Effects of the increased temperatures on the quantity of DNA in *recAts polA* cells
The number of chromosomes in AQ10549 cells was quantitatively measured using Pico Green, as shown in Fig. 2A. The relative number of chromosomes was determined using the mean FL-1A with the P3 gate. 30 °C (blue) and 42 °C (red) temperatures are shown, and each determination is presented as standard error of the mean (n ≥ 3, SEM). (**b**) Effects of the increased temperatures on anucleate cell production in *recAts polA* cells As in Fig. S2A, quantitative pico green staining was performed for anucleate cells in AQ10549 cells. The number of non-stained cell particles, that is, cells with less than one chromosome, in the particle fraction (P3 gate) of anucleate cells ($< 5 \times 10^4$ RFU) was determined. The percentage of anucleate cells in the particle fraction is shown as results for 30 °C (blue) and 42 °C (red), as well as standard error of the mean (n ≥ 3, SEM).

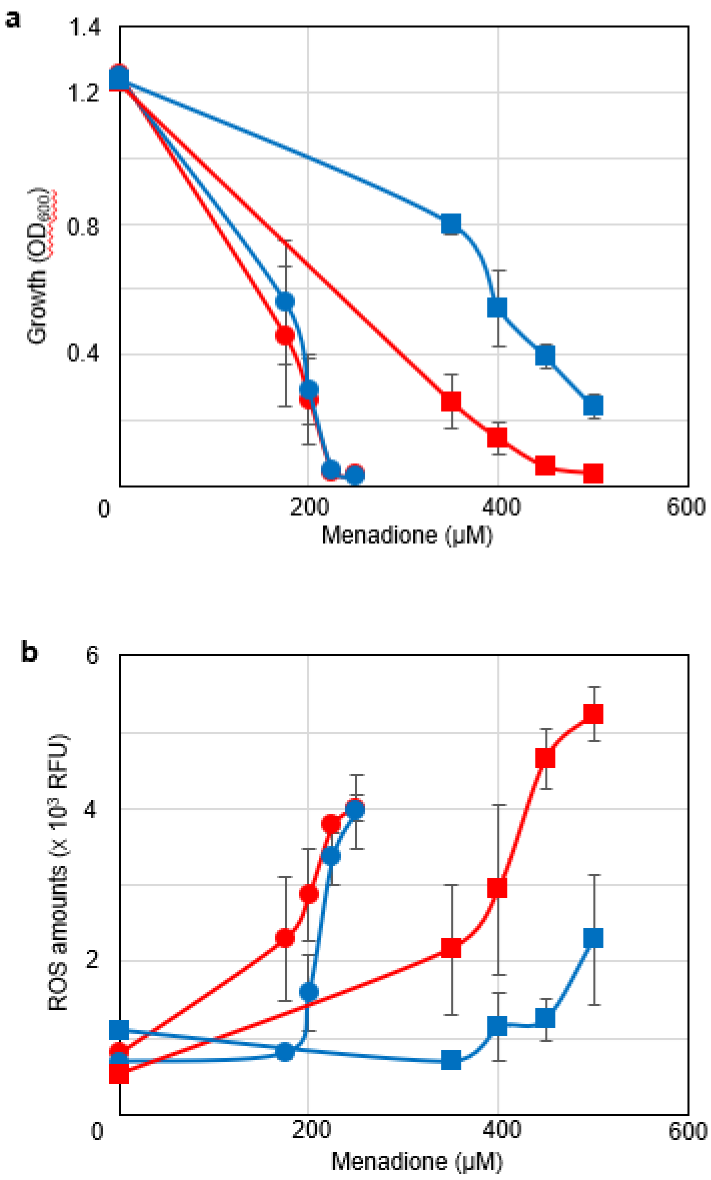

Fig. S5 Effect of *hslO* plasmids on the addition of menadione to *recAts polA* cells

(a) Suppression of growth inhibition with menadione by the *hslO* plasmid in *recAts polA* cells

The effects of the presence or the absence of the *hslO* plasmid on the growth of *recAts polA* cells inhibited by menadione were compared using inoculation experiments. Each determination was measured 16 h post cultivation. The *recAts polA* strain (AQ10549) is indicated using circles with the vector cells (TK3072) in red and the *hslO* plasmid cells (TK3075) in blue. *recAts polA ΔhslO lexA*(Def) cells are indicated using squares with the vector cells (TK4558) in red and the *hslO* plasmid cells (TK4559) in blue. Each determination is shown as standard error of the mean ($n \geq 3$, SEM).

(b) Suppression of ROS accumulation with menadione by a *hslO* plasmid in *recAts polA* cells

The effects of the presence or the absence of the *hslO* plasmid on the accumulation of ROS in the *recAts polA* cells treated with menadione were compared using inoculation experiments. Each determination was measured 16 h after inoculation, as in the previous experiments. The line graphs are shown as in Fig. 6a. Each determination is shown as standard error of the mean ($n \geq 3$, SEM).
